# Leisure-time physical activity, sun exposure, and multiple sclerosis risk: a population-based case–control study

**DOI:** 10.1007/s00415-026-14170-9

**Published:** 2026-09-26

**Authors:** Qingyuan Guo, Tomas Olsson, Lars Alfredsson, Anna Karin Hedström

**Affiliations:** 1 Department of Clinical Neuroscience, Center of Molecular Medicine, Karolinska Institutet, L8, 17176 Stockholm, Sweden; 2 Institute of Environmental Medicine, Karolinska Institutet, Stockholm, Sweden; 3 Centre for Occupational and Environmental Medicine, Region Stockholm, Stockholm, Sweden

**Keywords:** Multiple sclerosis, Physical activity, Sun exposure, MS risk

## Abstract

**Background:**

Physical activity benefits symptoms and function in people with multiple sclerosis (MS), but its relevance to MS susceptibility remains uncertain and may be difficult to separate from correlated lifestyle and environmental factors, including sun exposure.

**Methods:**

This Swedish population-based case–control study included 3,008 incident MS cases and 5773 matched controls. Analyses were restricted to participants whose MS onset, or corresponding reference year among controls, occurred within 5 years before study inclusion. Leisure-time physical activity 5 years before inclusion was classified into four levels. Odds ratios (ORs) with 95% confidence intervals (CIs) were estimated using logistic regression overall and by sun exposure. Time spent outdoors was additionally considered in a subgroup.

**Results:**

In models adjusted for age, sex, and residential area, the highest versus lowest level of physical activity was associated with lower MS risk (OR 0.78, 95% CI 0.67–0.92), but the association was attenuated after multivariable adjustment (OR 0.94, 95% CI 0.80–1.11). High physical activity was associated with lower risk among individuals with low sun exposure (adjusted OR 0.76, 95% CI 0.58–0.99), whereas no association was observed among those with high sun exposure (adjusted OR 0.97, 95% CI 0.78–1.20). In the subgroup, additional adjustment for outdoor activity had little influence on the estimates.

**Conclusion:**

The overall association between leisure-time physical activity and MS risk was attenuated after adjustment for lifestyle and environmental factors. Inverse associations among individuals with low sun exposure and in analyses accounting for outdoor activity suggest a possible context-dependent association.

**Supplementary Information:**

The online version contains supplementary material available at https://doi.org/10.1007/s00415-026-14170-9.

## Introduction

Multiple sclerosis (MS) is a chronic immune-mediated demyelinating disease and a major cause of neurological disability in young adults. MS susceptibility reflects a complex interplay between genetic predisposition and environmental exposures, several of which are potentially modifiable [1]. Physical activity is of interest in this context given its effects on immune regulation, inflammation, and metabolic health [2]. In people with established MS, physical activity has recognised benefits for symptoms and functional outcomes, including fatigue, mood, and quality of life [3–7]. Whether physical activity before disease onset is associated with the risk of developing MS remains less clear.

Previous studies of physical activity in relation to MS risk have reported inconsistent findings. EnvIMS and a Norwegian conscription cohort suggested inverse associations between physical activity or fitness and risk of MS [8, 9], whereas a large US prospective cohort found weaker evidence and raised the possibility of reverse causation [10]. Interpretation is complicated because physical activity clusters with other lifestyle and environmental factors, including smoking, body mass index, education, time spent outdoors, and sun exposure [11, 12].

In this study, we examined the association between leisure-time physical activity and MS risk, with particular attention to variation by sun exposure and adjustment for related lifestyle and environmental factors.

## Methods

The study was based on Environmental Investigation in Multiple Sclerosis (EIMS) which was designed as a population-based case–control study of MS etiology [13]. Eligibility criteria for cases were age 16–70 years, residence in Sweden, and a neurologist-confirmed diagnosis of MS according to the McDonald criteria [14]. All cases were recruited through hospital-based or private neurology units (a total of 40 recruiting centres, including all university hospitals). For each case, two controls were randomly selected from the national population register based on sex, age (5-year age groups), and residential area. A total of 3,940 cases and 7,339 controls were recruited between April 2005 and December 2019, with a response rate of 91% among cases and 71% among controls. The year of MS onset was defined as the index year for cases, and the same year was assigned to their matched controls. Cases and controls for whom five years or more had elapsed between the index year and study inclusion were excluded. We further excluded participants with missing information on covariates, resulting in an analytical sample of 3008 cases and 5773 controls. The participant selection process is shown in eFigure 1.

### Standard protocol approvals, registrations, and patient consents

The study was approved by the Regional Ethical Review Board at Karolinska Institute (reference number 2004–252-1:4) and conducted in accordance with the ethical principles outlined in the Declaration of Helsinki. All participants provided informed consents to participate and consent to publish.

### Assessment of physical activity

A self-administered questionnaire was used to collect comprehensive information on potential factors related to MS aetiology. At study inclusion, participants reported their leisure-time physical activity five years before inclusion. As the analyses were restricted to participants for whom less than 5 years had elapsed between the index year and study inclusion, the reported physical activity referred to a time before the index year. If physical activity varied substantially between seasons, participants were asked to report their average level across the year. The exposure was categorised into four predefined levels: low, moderate, moderate-high, or high physical activity. The same classification has previously been used in Swedish population-based research [15].

Low physical activity corresponded to mainly sedentary leisure-time activities with less than two hours per week of light exercise, such as walking or cycling. Moderate physical activity corresponded to at least 2 h of light exercise per week, usually without sweating, including active commuting and non-vigorous recreational activities (e.g. table tennis, bowling). Moderate-high physical activity corresponded to sweat-inducing exercise for at least 30 min one to two times per week. High physical activity corresponded to sweat-inducing exercise for at least 30 min three or more times per week.

### Assessment of covariates

Sun exposure during the 5-year period before inclusion was assessed using three questions regarding sunbathing in Sweden, travelling to sunnier countries, and use of sunbeds. Each answer alternative was given a number ranging from 1 (the lowest level) to 4 (the highest level), and the scores were summed to construct a composite index (range 3–12). Sun exposure was classified as low (< 6) versus high (≥ 6).

Ancestry was classified as Nordic or non-Nordic. Participants were categorised as Nordic if the participant and both parents were born within the Nordic countries. Educational level was classified into university studies versus no university studies. Based on smoking habits before and at the index year, participants were categorised as current smokers, past smokers, or never smokers. Alcohol use was reported as weekly intake of beer, wine, strong wine, and liquor, and converted to grams/week. Body mass index (BMI) at age 20 years. BMI at age 20 was calculated from self-reported weight at age 20 and current height and categorised as normal weight (< 25 kg/m^2^), overweight (25–30 kg/m^2^) and obesity (> 30 kg/m^2^).

For participants recruited after 2015 (851 cases and 1,143 controls), outdoor exposure was assessed using two items covering time spent outdoors during summer leisure and outdoor exposure at work. Both items were rated on a four-point ordinal scale ranging from never to several hours per day. The two items were summed to generate a composite outdoor exposure score, with higher values indicating greater overall time spent outdoors.

### Statistical analysis

Associations between physical activity and risk of MS were assessed using logistic regression models, with odds ratios (ORs) and 95% confidence intervals (CIs). Physical activity was initially modelled categorically using four predefined levels reflecting increasing leisure-time physical activity intensity, with the lowest level as the reference category. Trends across physical activity levels were evaluated by modelling the variable as an ordinal continuous term. We further performed stratified analyses to estimate the association between physical activity and MS risk separately among individuals with lower and higher levels of sun exposure. For stratified analyses, sun exposure was dichotomized into low and high levels using the median sun exposure index (6) as the cut-off.

Correlations between physical activity and demographic, lifestyle-related, and environmental covariates were assessed using Spearman correlation coefficients. To assess whether associations between physical activity and MS risk varied according to these covariates, multiplicative interactions were evaluated by including product terms between physical activity and each respective factor in multivariable logistic regression models. Physical activity was modelled as an ordinal continuous variable, with ORs expressed per one-level increase. Continuous variables were modelled per unit increase. Statistical evidence for interaction was assessed using Wald tests.

To further examine the interaction between physical activity and sun exposure, the sun exposure index was categorised as lower (< 6), intermediate (= 6), or higher (> 6). Physical activity was modelled as an ordinal variable, with ORs expressed per one-level increase. Product terms between physical activity and the three-level sun exposure variable were included in the multivariable logistic regression model, and statistical evidence for interaction was assessed using a global two-degree-of-freedom Wald test. From the interaction model, ORs for a one-level increase in physical activity were estimated separately within each sun exposure category. Model-predicted probabilities of MS were estimated across the four physical activity levels from the same model and used for graphical presentation.

A minimally adjusted model included age, sex, and residential area. The multivariable model further included ancestry, educational level, smoking status, alcohol consumption, BMI status, and sun exposure. In a subgroup with available data on time spent outdoors, we performed sensitivity analyses in which physical activity was modelled as an ordinal variable, with ORs expressed per one-level increase. Analyses were conducted overall and stratified by sun exposure, with additional adjustment for outdoor exposure, including time spent outdoors during summer leisure and outdoor occupational exposure. Finally, to assess potential residual confounding by population structure, we repeated the ordinal trend analysis among participants with available genetic data, replacing Nordic origin with adjustment for six genetic principal components. All statistical analyses were performed using SAS software version 9.4 (SAS Institute Inc., Cary, NC, USA). Two-sided p-values <0.05 were considered statistically significant.

## Results

The study comprised 3,008 cases and 5,773 matched controls. The mean age at diagnosis among cases was 36.3 years (SD=10.8). Individuals reporting higher physical activity tended to have a more favourable lifestyle profile compared to those with lower physical activity. In both cases and controls, higher physical activity was associated with higher educational attainment, a lower prevalence of smoking and higher sun exposure. Characteristics of cases and controls according to physical activity level are presented in Table 1.

**Table 1:** Characteristics of study participants, by physical activity level

Characteristics	Total		Physical activity level 1–2		Physical activity level 3–4	
	Cases n=3008	Controls, n=5773	Cases, n=1436	Controls, n=2536	Cases, n=1572	Controls, n=3237
Age at index (SD)	35.2 (10.7)	34.8 (10.8)	36.1 (10.7)	35.8 (10.8)	34.4 (10.6)	34.1 (10.8)
Age at diagnosis (SD)	36.3 (10.8)	–	37.2 (10.8)	–	35.5 (10.8)	–
Age at study inclusion (SD)	36.7 (10.8)	36.2 (11.0)	37.6 (10.8)	37.2 (11.1)	35.9 (10.8)	35.4 (10.9)
Female, n (%)	2136 (71.0)	4137 (71.7)	1015 (70.7)	1864 (73.5)	1121 (71.3)	2273 (70.2)
Nordic, n (%)	2447 (81.4)	4604 (79.8)	1140 (79.4)	1945 (76.7)	1307 (83.1)	2659 (82.1)
First recorded EDSS (IQR)	1.5 (0–2.5)	–-	1.5 (0–2.5)	–	1.5 (0–2.5)	–
University studies, n (%)	1371 (45.6)	2370 (41.1)	567 (39.5)	923 (36.4)	804 (51.2)	1447 (44.7)
Never smokers, n (%) Current smokers, n (%) Past smokers, n (%)	1458 (48.5) 725 (24.1) 825 (27.4)	3356 (58.1) 1196 (20.7) 1221 (21.2)	607 (42.3) 413 (28.8) 416 (29.0)	1379 (54.4) 596 (23.5) 561 (22.1)	851 (54.1) 312 (19.9) 409 (26.0)	1977 (61.1) 600 (18.5) 660 (20.4)
Alcohol use, n (%)	1984 (66.0)	3979 (68.9)	903 (62.9)	1644 (64.8)	1081 (68.8)	2335 (72.1)
Alcohol, g/week (IQR)	40 (24–75)	48 (30–87)	44 (24–80)	48 (28–90)	39 (24–73)	48 (30–84)
BMI at age 20, kg/m^2^ (SD)	21.8 (6.4)	21.1 (5.8)	22.1 (6.5)	21.2 (6.1)	21.6 (6.2)	21.1 (5.5)
Sun exposure index (SD)	6.2 (1.8)	6.5 (1.9)	5.8 (1.8)	6.2 (1.9)	6.5 (1.7)	6.8 (1.9)

*EDSS* expanded disability status scale, *SD* standard deviation, *IQR* interquartile range. Physical activity levels 1–2 represent non–sweat-inducing leisure-time physical activity, whereas levels 3–4 represent regular sweat-inducing physical activity. Index refers to the year of disease onset for cases and the corresponding matched index year for controls

Physical activity was correlated with several lifestyle and environmental covariates (eTable 1). The strongest correlations were observed for outdoor time during summer leisure (r=0.27, p<0.0001) and sun exposure (r=0.19, p<0.0001). Among the examined covariates, only sun exposure showed statistical evidence of multiplicative interaction with physical activity in relation to MS risk (p=0.01).

In the minimally adjusted model, higher levels of physical activity were associated with lower odds of MS (OR per level 0.92, 95% CI 0.87–0.96) (Table 2). However, after additional adjustment, the trend was attenuated (OR per level 0.97, 95% CI 0.93–1.02), and no clear overall association between physical activity and MS risk remained. The estimate for high versus low physical activity moved towards the null, from 0.78 (95% CI 0.67–0.92) in the minimally adjusted model to 0.94 (95% CI 0.80–1.11) in the multivariable model.

**Table 2:** Associations between physical activity levels and risk of MS

Physical activity	Cases/controls	OR (95% CI)^1^	OR for trend	OR (95% CI)^1–2^	OR for trend
Low	363/642	1.0 (reference)	0.92 (0.87–0.96)	1.0 (reference)	0.97 (0.93–1.02)
Moderate	1073/1894	0.99 (0.85–1.15)		1.04 (0.89–1.22)	
Moderate-high	871/1675	0.91 (0.78–1.06)		1.02 (0.87–1.19)	
High	701/1562	0.78 (0.67–0.92)		0.94 (0.80–1.11)	
High sun exposure					
Low	169/372	1.0 (reference)	0.96 (0.91–1.02)	1.0 (reference)	1.00 (0.95–1.06)
Moderate	597/1178	1.10 (0.91–1.38)		1.13 (0.92–1.39)	
Moderate-high	590/1181	1.09 (0.90–1.35)		1.12 (0.91–1.38)	
High	524/1204	0.96 (0.78–1.18)		0.97 (0.78–1.20)	
Low sun exposure					
Low	194/270	1.0 (reference)	0.88 (0.81–0.95)	1.0 (reference)	0.91 (0.84–0.98)
Moderate	476/716	0.91 (0.73–1.14)		0.95 (0.76–1.18)	
Moderate-high	281/494	0.79 (0.62–1.00)		0.84 (0.66–1.08)	
High	177/358	0.68 (0.53–0.89)		0.76 (0.58–0.99)	

ORs for trend represent the association per increasing physical activity level

^1^Adjusted for age, sex, residential area

^2^Adjusted for educational level, ancestry, smoking status, alcohol consumption, BMI status, and sun exposure

**Fig. 1:**
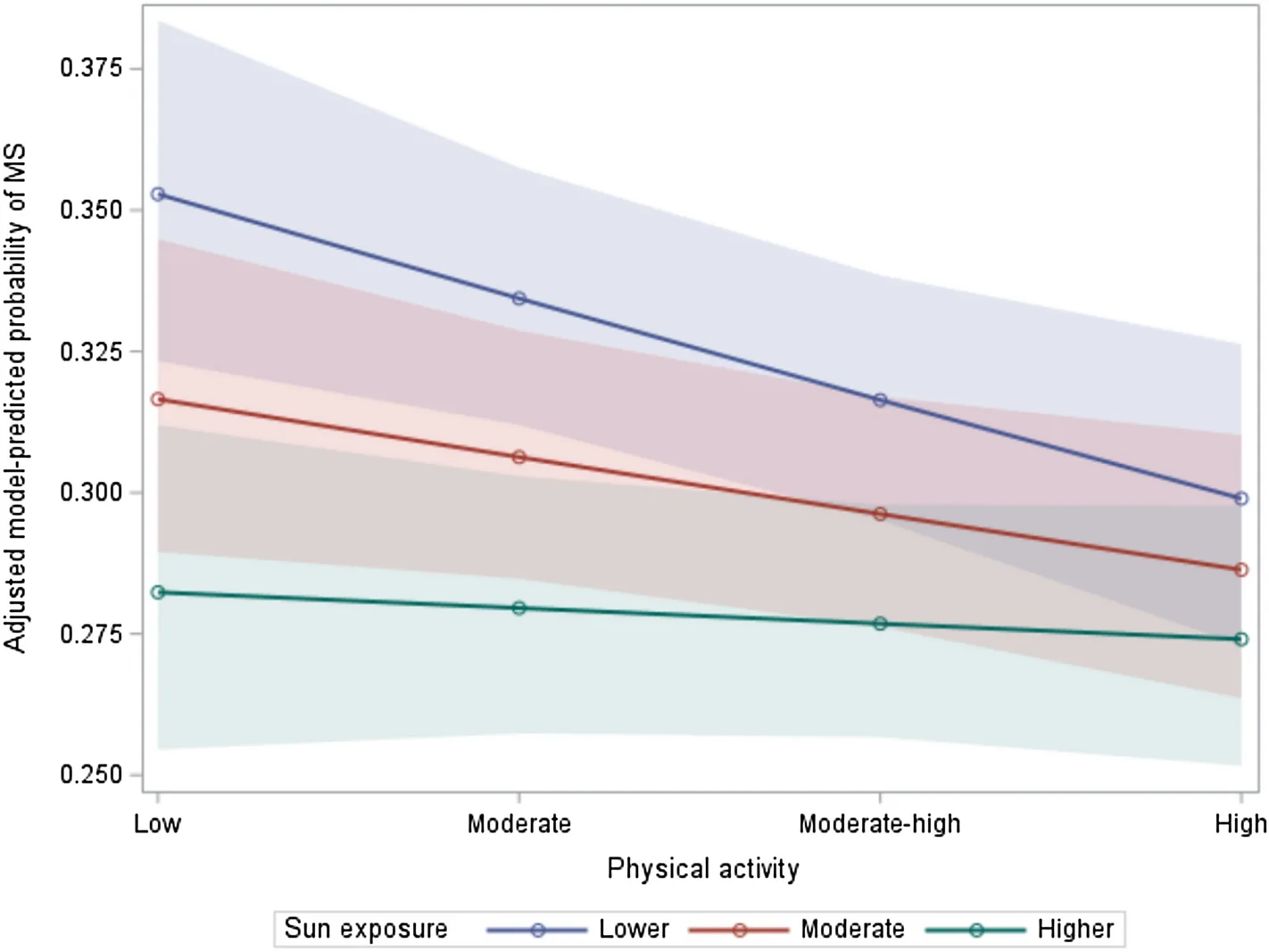
Model-predicted probabilities of MS according to physical activity level and sun exposure. Predicted probabilities were estimated from a multivariable logistic regression model including physical activity as an ordinal variable, sun exposure categorised as <6, 6, or >6, and their interaction. Lines represent adjusted model-predicted probabilities of MS, and shaded areas represent 95% confidence intervals. The model was adjusted for age, sex, residential area, ancestry, educational level, smoking status, alcohol consumption, and BMI status. Predictions are shown at each of the four physical activity levels and within each sun exposure category

When analyses were stratified by sun exposure, the inverse association between physical activity and MS risk was apparent among participants with lower sun exposure. In this stratum, the adjusted OR for the highest versus lowest physical activity level was 0.76 (95% CI 0.58–0.99), with an inverse trend across activity levels. No corresponding patterns were observed among participants with high sun exposure (Table 2). This pattern was also reflected in the interaction model, where the estimated association of physical activity with MS risk was strongest at low sun exposure and attenuated at higher levels of sun exposure (Table 3). The same gradient was illustrated by the model-predicted probabilities in Fig. 1.

**Table 3:** Associations between leisure-time physical activity and MS risk according to sun exposure

Sun exposure index	OR (95% CI)	P value
<6	0.88 (081–0.95)	0.002
6	0.95 (0.86–1.05)	0.33
>6	1.01 (0.94–1.09)	0.71

Odds ratios represent the association between a one-level increase in physical activity and MS risk within each sun exposure category. Estimates were derived from a multivariable logistic regression model including physical activity, sun exposure categorised as <6, 6, or >6, and their interaction. The model was adjusted for age, sex, residential area, educational level, ancestry, smoking status, alcohol consumption, and BMI status

Among participants with available genetic data, the OR per one-level increase in physical activity was 0.96 (95% CI 0.87–1.06) after adjustment for Nordic origin and 0.96 (95% CI 0.88–1.06) after adjustment for genetic principal components. In the subgroup with available information on leisure-time and occupational outdoor activity, higher physical activity was associated with lower odds of MS in the adjusted model (OR per one-level increase 0.87, 95% CI 0.79–0.96). The estimate changed little after additional adjustment for time spent outdoors (OR per one-level increase 0.88, 95% CI 0.80–0.98), and similar estimates were observed in analyses stratified by sun exposure (Table 4).

**Table 4:** Sensitivity analysis further adjusted for outdoor exposure: associations between physical activity levels and risk of MS

Physical activity	Cases/controls	OR (95% CI)^1^	OR (95% CI)^1–2^	OR (95% CI)^1–3^
	851/1143	0.81 (0.74-0.88)	0.87 (0.79-0.96)	0.88 (0.80-0.98)
High sun exposure				
Physical activity	Cases/controls	OR (95% CI)^1^	OR (95% CI)^1–2^	OR (95% CI)^1–3^
	525/751	0.83 (0.71–0.97)	0.88 (0.77–0.99)	0.87 (0.74–1.03)
Low sun exposure				
Physical activity	Cases/controls	OR (95% CI)^1^	OR (95% CI)^1–2^	OR (95% CI)^1–3^
	326/392	0.80 (0.71–0.90)	0.83 (0.71–0.98)	0.88 (0.77–1.00)

^1^Adjusted for age, sex, residential area

^2^Adjusted for educational level, ancestry, smoking status, alcohol consumption, BMI status, and sun exposure

^3^Adjusted for leisure time spent outdoors in summer, and time spend outdoors at work

## Discussion

In this population-based case–control study, the inverse association between leisure-time physical activity and MS risk observed in minimally adjusted analyses was attenuated after adjustment for lifestyle and environmental factors. The remaining inverse association was most apparent among individuals with low sun exposure, with little evidence of an association at higher levels of sun exposure. In the subgroup with information on leisure-time and occupational outdoor activity, the estimates changed little after additional adjustment for time spent outdoors. This pattern suggests that the association between physical activity and MS risk may vary according to sun exposure, although the findings require cautious interpretation.

Previous observational studies of physical activity and MS risk have yielded mixed findings, with inverse associations in some studies and weaker evidence in others, particularly after accounting for potential confounding and reverse causation [8–10]. Complementing these observational findings, a Mendelian randomisation study reported inverse associations between genetically predicted moderate and overall physical activity and MS risk [16]. However, these genetically predicted measures are not directly comparable with self-reported leisure-time physical activity. Differences in study design, exposure timing, physical activity assessment, and covariate adjustment may contribute to variation across studies. Our overall findings align most closely with observational studies in which the association was attenuated after multivariable adjustment.

The variation by sun exposure is central to the interpretation of our findings. Physical activity was associated with lower MS risk mainly among individuals with low sun exposure, and this pattern was supported by the interaction analyses. This could reflect genuine effect modification by sun exposure, but it could also arise from imperfect measurement of sun exposure and outdoor behaviour, or because physical activity captures differences in health status, socio-economic patterning, and other correlated lifestyle factors. The limited change after adjustment for measured outdoor activity argues against time spent outdoors as the sole explanation but does not establish an independent effect of physical activity.

Several mechanisms could plausibly link physical activity to MS susceptibility. Regular physical activity has systemic anti-inflammatory effects and may influence cytokine profiles, metabolic regulation, adiposity-related inflammation, and myokine signalling [5, 17]. These pathways are relevant to immune processes implicated in CNS autoimmunity. However, most mechanistic evidence comes from experimental studies or studies of people with established MS, and its relevance to disease initiation remains uncertain. Physical activity may also be a marker of other favourable health behaviours, making it difficult to separate biological effects of activity itself from correlated environmental and lifestyle exposures.

The main strengths of this study include its population-based design and the large number of incident MS cases. The study also benefits from a high participation rate and comprehensive exposure data, which enabled adjustment for multiple potential confounders.

Several limitations should be acknowledged. Lifestyle and environmental exposures were self-reported retrospectively and recall bias cannot be excluded. Although restricting the analyses to participants whose index year occurred less than 5 years before inclusion ensured that the reported physical activity preceded the index year, the interval between physical activity assessment and clinical onset varied and could be short. Reverse causation therefore remains an important concern, as MS may be preceded by a prodromal phase during which fatigue, reduced exercise tolerance, pain, mood changes, or subtle neurological dysfunction may influence physical activity before clinical onset [18, 19]. Thus, although the design reduces some sources of bias, it cannot ensure that reported physical activity preceded all disease-related behavioural change.

The broad physical activity categories may not capture all relevant aspects of activity, including duration, intensity, frequency, seasonal variation, or changes over time. Similarly, the sun exposure index and outdoor activity variables may be imperfect measures of relevant sun-related behaviours and total ultraviolet radiation exposure. If physical activity influenced sun-related or outdoor behaviours, adjustment for these factors may have attenuated part of the association. Since several potential effect modifiers were examined and the subgroup estimates were imprecise, the interaction with sun exposure should be considered exploratory and requires replication. Residual confounding by other unmeasured or imperfectly measured factors, such as diet, vitamin D supplementation, comorbidity, occupational conditions, or other health-related behaviours, may have influenced the results. Finally, selection bias cannot be excluded. However, the distributions of several lifestyle characteristics, including smoking and BMI, among EIMS controls were broadly similar to population-based data from Statistics Sweden for corresponding age groups [20]. As comparable population data were unavailable for physical activity and sun exposure, the representativeness of the controls with respect to these exposures could not be directly assessed.

In conclusion, leisure-time physical activity was not clearly associated with MS risk in the overall multivariable analysis, but an inverse association was observed among individuals with low sun exposure and in sensitivity analyses with adjustment for outdoor activity. The results are compatible with a modest inverse association in some exposure contexts and highlight the importance of considering physical activity in relation to sun exposure, outdoor activity, and broader lifestyle patterns.

## Supplementary Information

Below is the link to the electronic supplementary material.

Supplementary Material 1.

## Data Availability

Anonymized data underlying this article will be made available upon reasonable request to qualified investigators, subject to applicable ethical and data-protection requirements.
