# Supplementary material for: Leisure-time physical activity, sun exposure, and multiple sclerosis risk: a population-based case–control study
Source: J Neurol. 2026 Sep 26;273(10):623. doi: 10.1007/s00415-026-14170-9 (PMC13615984; doi:10.1007/s00415-026-14170-9)
Supplement: Supplementary file 1 [file 415_2026_14170_MOESM1_ESM.docx]

eFigure 1. Flowchart of participant selection and study inclusion

eTable 1. Correlations between physical activity and selected covariates, and interaction analyses in relation to MS risk

| Factor | Correlation with physical activity (r, p-value) | Interaction p-value |
| --- | --- | --- |
| Nordic ancestry | 0.08, <0.0001 | 0.24 |
| Education | 0.12, <0.0001 | 0.43 |
| Smoking status | -0.07, <0.0001 | 0.14 |
| BMI status | -0.07, <0.0001 | 0.47 |
| Alcohol consumption | 0.08, <0.0001 | 0.57 |
| Sun exposure | 0.19, <0.0001 | **0.01** |
| Outdoor time at work | 0.05, 0.02 | 0.83 |
| Outdoor time during summer leisure | 0.27, <0.0001 | 0.12 |

Interaction p-values were derived from multivariable logistic regression models including physical activity, the respective lifestyle-related factor, and their interaction term, adjusted for age, sex, residential area, ancestry, educational level, smoking status, alcohol consumption, BMI status, and sun exposure.

eTable 2. Characteristics of study participants with outdoor-activity data.

| Characteristics | Total | | Physical activity level 1-2 | | Physical activity level 3-4 | |
| --- | --- | --- | --- | --- | --- | --- |
|  | Cases n=851 | Controls, n=1143 | Cases, n=426 | Controls, n=468 | Cases, n=425 | Controls, n=675 |
| Age at index (SD) | 35.0 (10.7) | 32.9 (11.1) | 35.1 (10.4) | 33.3 (11.2) | 34.9 (11.0) | 32.6 (11.1) |
| Age at diagnosis (SD) | 36.0 (10.7) | - | 36.1 (10.3) | - | 35.9 (11.1) | - |
| Age at study inclusion (SD) | 36.3 (10.9) | 35.8 (11.1) | 36.3 (10.5) | 35.3 (11.2) | 36.3 (11.2) | 34.7 (10.5) |
| Female, n (%) | 591 (69.5) | 822 (71.9) | 297 (69.7) | 344 (73.5) | 294 (69.2) | 478 (70.8) |
| Nordic, n (%) | 705 (82.8) | 1000 (87.5) | 340 (79.8) | 394 (84.2) | 365 (85.9) | 606 (89.8) |
| University studies, n (%) | 380 (44.7) | 539 (47.2) | 165 (38.7) | 189 (40.4) | 215 (50.6) | 350 (51.9) |
| First recorded EDSS (IQR) | 1.5 (0-2.0) | - | 1.5 (0-2.0) | - | 1.0 (0-2.0) | - |
| Never smokers, n (%)  Current smokers, n (%)  Past smokers, n (%) | 432 (50.8) 116 (13.6) 303 (35.6) | 740 (64.7) 168 (14.7) 235 (20.6) | 195 (45.8) 67 (15.7) 164 (38.5) | 295 (63.0) 73 (15.6) 100 (21.4) | 237 (55.8) 49 (11.5) 139 (32.7) | 445 (65.9) 95 (14.1) 135 (20.0) |
| Alcohol use, n (%) | 540 (63.5) | 715 (62.6) | 251 (58.9) | 267 (57.1) | 289 (68.0) | 448 (66.4) |
| Alcohol, gram/week (IQR) | 36 (24-72) | 36 (24-72) | 36 (24-72) | 36 (24-72) | 24 (24-60) | 48 (24-72) |
| BMI at age 20, kg/m^2^ (SD) | 22.7 (5.8) | 21.9 (5.5) | 22.8 (6.0) | 22.1 (6.1) | 22.6 (5.6) | 21.8 (5.1) |
| Sun exposure index (SD) | 6.1 (1.7) | 6.4 (1.7) | 5.7 (1.6) | 6.0 (1.7) | 6.5 (1.7) | 6.8 (1.7) |
| Outdoor exposure score (SD) | 5.1 (1.2) | 5.2 (1.2) | 4.9 (1.3) | 5.1 (1.3) | 5.2 (1.2) | 5.3 (1.2) |

Physical activity levels 1–2 represent non–sweat-inducing leisure-time physical activity, whereas levels 3–4 represent regular sweat-inducing physical activity. Index refers to the year of disease onset for cases and the corresponding matched index year for controls. EDSS=expanded disability status scale; SD=standard deviation; IQR=interquartile range.
